# A Chimeric Pneumovirus Fusion Protein Carrying Neutralizing Epitopes of Both MPV and RSV

**DOI:** 10.1371/journal.pone.0155917

**Published:** 2016-05-25

**Authors:** Xiaolin Wen, Jennifer Pickens, Jarrod J. Mousa, George P. Leser, Robert A. Lamb, James E. Crowe, Theodore S. Jardetzky

**Affiliations:** 1 Department of Structural Biology, Stanford University School of Medicine, Stanford, CA, United States of America; 2 Department of Pediatrics, Vanderbilt University School of Medicine, Nashville, TN, United States of America; 3 Monroe Carell Jr. Children's Hospital at Vanderbilt University, Nashville, TN, United States of America; 4 Howard Hughes Medical Institute, Northwestern University, Evanston, IL, United States of America; 5 Department of Molecular Biosciences, Northwestern University, Evanston, IL, United States of America; 6 Department of Pathology, Microbiology and Immunology, Vanderbilt University School of Medicine, Nashville, TN, United States of America; University of Georgia, UNITED STATES

## Abstract

Respiratory syncytial virus (RSV) and human metapneumovirus (HMPV) are paramyxoviruses that are responsible for substantial human health burden, particularly in children and the elderly. The fusion (F) glycoproteins are major targets of the neutralizing antibody response and studies have mapped dominant antigenic sites in F. Here we grafted a major neutralizing site of RSV F, recognized by the prophylactic monoclonal antibody palivizumab, onto HMPV F, generating a chimeric protein displaying epitopes of both viruses. We demonstrate that the resulting chimeric protein (RPM-1) is recognized by both anti-RSV and anti-HMPV F neutralizing antibodies indicating that it can be used to map the epitope specificity of antibodies raised against both viruses. Mice immunized with the RPM-1 chimeric antigen generate robust neutralizing antibody responses to MPV but weak or no cross-reactive recognition of RSV F, suggesting that grafting of the single palivizumab epitope stimulates a comparatively limited antibody response. The RPM-1 protein provides a new tool for characterizing the immune responses resulting from RSV and HMPV infections and provides insights into the requirements for developing a chimeric subunit vaccine that could induce robust and balanced immunity to both virus infections.

## Introduction

Respiratory syncytial virus (RSV) and human metapneumovirus (HMPV) are respiratory viruses that cause widespread morbidity within the human population second only to influenza virus [1]. RSV is the most common cause of bronchiolitis and pneumonia in infants worldwide and also impacts the elderly and others with weakened immune systems, leading to hundreds of thousands of hospitalizations and millions of hospital visits yearly in the US [2–4]. HMPV is a related virus first identified in 2001 [5] and is thought to have been causing respiratory illnesses in the human population for over 50 years. Similar to RSV, HMPV infections are associated with a significant burden of hospitalizations and hospital visits in young children, as well as in the elderly [6, 7]. There are no vaccines or small molecule antiviral treatments specific to these two viruses currently available. However, a humanized murine monoclonal antibody (palivizumab) has been developed for the prophylactic treatment of infants at high risk for RSV infection [8, 9]. In addition, a second generation, affinity-matured variant of palivizumab has been generated (motavizumab) [10, 11], although it has not been approved for therapeutic use.

**Fig 1:**
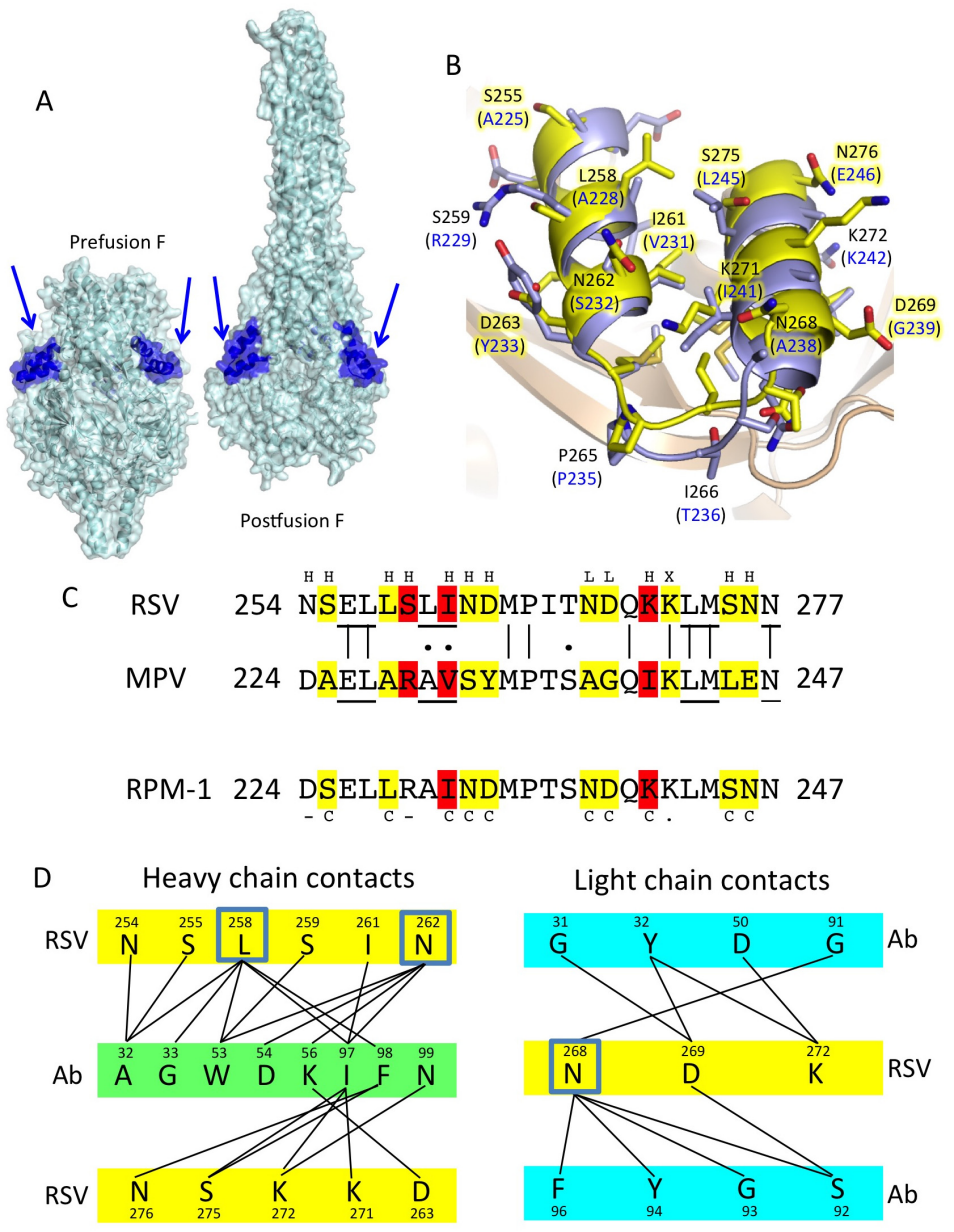
Grafting of the RSV palavizumab/motavizumab epitope onto the HMPV F protein. (a) Structures of pre- and post-fusion RSV F highlighting the helix-turn-helix motif for palivizumab and motavizumab. The protein surface and ribbon are colored cyan with the epitope region in each subunit of the trimer colored blue (arrows). (b) Structure of the motavizumab:RSV peptide complex (RCSB code: 3IXT) superimposed on the partial HMPV F structure (RCSB code: 4DAG). Residues from RSV are labeled in black and those of HMPV F are in parentheses and labeled in blue. Residues highlighted with the yellow background were substituted in the RPM-1 mutant. (c) Sequence alignment of RSV and HMPV F proteins, spanning the motavizumab epitope, along with the RPM-1 mutant sequence. Yellow and red boxes highlight amino acids that contact motavizumab, with red indicating less accessible residues of the epitope. Letters above (H/L) the sequences represent contacts with the heavy of light chain CDRs of the motavizumab Fab. Letters below the RPM-1 mutant represent contact residues that were either changed (C), left unchanged (-) or are identical (.) in RSV and HMPV F. (d) Contact map of motavizumab:RSV F peptide contacts from the crystal structure, with RSV residues highlighted in yellow and antibody residues highlighted in green (heavy chain) or blue (light chain). Three residues (L258, N262, N268) are highlighted as making the most contacts per residue across the interface.

RSV and HMPV belong to the larger paramyxovirus family, which includes mumps virus, measles virus and human parainfluenza viruses [1, 12]. RSV and HMPV are the two defining viruses of the pneumovirinae subset of the larger family [1, 12]. The paramyxoviruses are segmented, negative strand RNA viruses, which bud from cells as lipid enveloped viral particles. Most paramyxoviruses encode ~9 proteins and express two glycoproteins embedded in the particle envelope that are responsible for host cell binding, membrane fusion and virus entry [12, 13]. RSV and HMPV produce an attachment protein (G) and fusion (F) protein, which mediate these functions and which are also targets of the immune response. The G protein is a type II membrane protein, with highly glycosylated, mucin-like domains, as well as a CX3C chemokine-like motif that may be involved in modulating immune responses during infection [1]. The F protein is a member of the class I viral fusion proteins and it undergoes large conformational changes during virus entry to promote viral and cellular membrane fusion (Fig 1A) [13–18]. While F and G both generate antibodies during the immune response, the F protein is the major target of the neutralizing antibody (nAb) response to infection and substantial effort has been invested in understanding the mechanisms of nAb binding specificity. The sites for major neutralizing epitopes on F have been mapped, including a prominent helix-turn-helix motif that is recognized by palivizumab and motavizumab (designated site II or site A in a second nomenclature) [19]. HMPV F sites fall into 6 groups, 5 of which have been mapped by selecting mAb-resistant virus mutants [20–22].

The structure of motavizumab in complex with a peptide from RSV F spanning residues 254–277 has been determined [19] and recently a low-resolution complex with RSV F has also been solved [23], demonstrating that this epitope forms a helix-turn-helix structural motif. In addition, the structure of an anti-HMPV F antibody (DS7) in complex with a fragment of the MPV F protein revealed a novel neutralizing site within a beta-sheet domain of F (domain I; D-I). Two antibodies specific for the pre-fusion conformation of RSV F (D25 and AM14) were isolated and structures with pre-fusion RSV F determined, revealing epitopes located in the conformationally labile head region of F [15, 23]. Despite considerable sequence variation between RSV and HMPV F proteins (36.7% identity), antibodies capable of neutralizing both RSV and HMPV have been identified, raising the possibility that a single vaccine antigen could be developed to protect against both virus infections [24, 25].

Efforts to develop a vaccine against RSV have met with numerous complications including a failed formalin-treated vaccine trial in the 1960s, which induced more severe disease in vaccinated children rather than the sought after protection [1]. Approaches to RSV vaccine development have included the production of attenuated or otherwise engineered viruses, as well as a variety of F antigen preparations. Recently, stabilized forms of the pre-fusion F trimer have been engineered to overcome issues with the inherent conformational ability of RSV F and to produce a potentially more potent subunit-based vaccine [26–28]. Both pre- and post-fusion conformations of RSV F protein provide protective immune response to virus in animal studies, although pre-fusion F may elicit a stronger neutralizing response [17, 24, 27]. It is unclear how these responses might be related to the previously observed vaccine-induced disease. As an alternative to RSV F based vaccines, epitope scaffolding approaches also have shown promise in stimulating highly targeted neutralizing antibody responses to RSV [29–31]. The helix-turn-helix motif defining antigenic site II or A that is recognized by palivizumab/motavizumab has been reconstructed computationally onto an unrelated helical protein backbone, and this engineered antigen has been shown to induce an RSV neutralizing antibody response in macaques but not in mice [29]. The site A epitope also has been inserted into an immunoglobulin scaffold, resulting in an immunogen that stimulates neutralizing RSV antibodies in mice [31]. Although these studies show progress towards developing an effective and safe RSV vaccine, many questions remain regarding the nature of the antibodies generated in natural and vaccine-induced responses as well as in the breadth of protection and potential for vaccine-induced disease in humans.

Here we generated a novel chimeric F protein mutant to investigate its properties as a potential probe for the specificity of immune responses to RSV and HMPV, as well as a potential vaccine antigen that could induce protective immune responses to both viruses. We grafted residues of the RSV site A epitope onto the HMPV F protein. We show that the chimeric protein expresses well and adopts pre- and post-fusion conformations similar to those of wild-type HMPV F. The resulting MPV/RSV chimeric protein, referred to as RPM-1, retained neutralizing epitopes derived from both viruses. The RPM-1 mutant, but not wild-type HMPV F, was bound by both palivizumab and motavizumab with nanomolar and picomolar affinities, respectively. The chimeric F mutant also retained its binding to the anti-HMPV F nAb DS7. The RPM-1 protein can be used to characterize the specificity of anti-RSV and anti-HMPV antibodies, mapping the reactivity to the palivizumab site. Finally, we demonstrated that immunization of mice with RPM-1 induces serum neutralizing antibody responses to MPV, but not RSV, demonstrating that the presence of the grafted RSV site A epitope within MPV F is comparatively ineffective at stimulating a robust antibody response. Nonetheless, this chimeric HMPV/RSV F approach provides a foundation for further developing a single vaccine antigen that could provide protection against both RSV and HMPV infections.

## Results

### Design and expression of a chimeric RSV/HMPV F protein

The motavizumab/palivizumab (M/P) epitope encompasses a helix-turn-helix motif in RSV F that is displayed as ridge lying above the surrounding F protein surface. It is well exposed in both the pre- and post-fusion forms of the RSV F protein (Fig 1A). The structures of motavizumab in complex with RSV peptide (residues 254 to 277), RSV F [23] and a scaffolded variant have been determined [19, 29]. The antibody stabilizes the native conformation of the free peptide (Fig 1B) into the helix-turn-helix motif that is nearly identical to the conformation observed in the native RSV and HMPV F proteins. Structural superposition of the analogous residues in HMPV F (amino acids 224 to 247) shows very good correspondence between the two motifs, despite relatively low amino acid conservation (Fig 1B). Over the 24 epitope residues, 9 are identical between RSV and HMPV, giving ~24% identity between the two proteins (Fig 1C). Although the structural correspondence is good (Fig 1B), differences in the relative positions of the two helices and the intervening connecting loop are evident in the superposition of the two structures (Fig 1B).

Motavizumab makes direct contacts with 13 of the 24 epitope residues within the peptide (Fig 1C and 1D). Of the contact residues, only one is conserved between RSV and HMPV sequences (K272), consistent with the specificity of motavizumab for RSV over MPV F. The conserved residues (E256, L257, M264, P265, Q270, K272, L273, M274 and N277) are predominantly buried, forming the structural base of the surface displayed epitope.

Based on the high conservation of the buried residues of the motavizumab epitope between RSV and HMPV, substitution of the surface residues of the motif seemed unlikely to disrupt the MPV structure and would result in the grafting of the epitope from RSV onto the HMPV F protein. In designing the chimeric HMPV mutant, two of the 12 nonconserved motavizumab contact residues (N254 and S259) were not substituted to the corresponding RSV amino acid. The side chains of N254 and S259 appeared less important for antibody binding. For example, although S259 participates in the motavizumab interface, it makes a single contact with the antibody through its main chain. The corresponding residue in HMPV is R229 (Fig 1B), and it makes hydrogen bonds within DIII that could be important for stabilizing the pre-fusion HMPV F structure [14], therefore this HMPV residue was retained in the chimeric mutant. The remaining 10 motavizumab contact residues appeared more critical for epitope definition (S255, L258, I261, N262, D263, N268, D269, K271, S275 and N276). In particular, residues L258, N262 and N268 were observed to make 14 contacts to multiple motavizumab residues at the center of the interface (Fig 1D, boxed) and these residues are very different in MPV (A, S, A, respectively, Fig 1C). Residues D269, K272 and S275 also make a total of seven contacts to motavizumab that are also centrally located in the interface (Fig 1B and 1D). The final sequence of the chimeric protein generated, referred to as RPM-1, is shown in Fig 1C.

### The RPM-1 mutant adopts conformations similar to wild-type HMPV F

**Fig 2:**
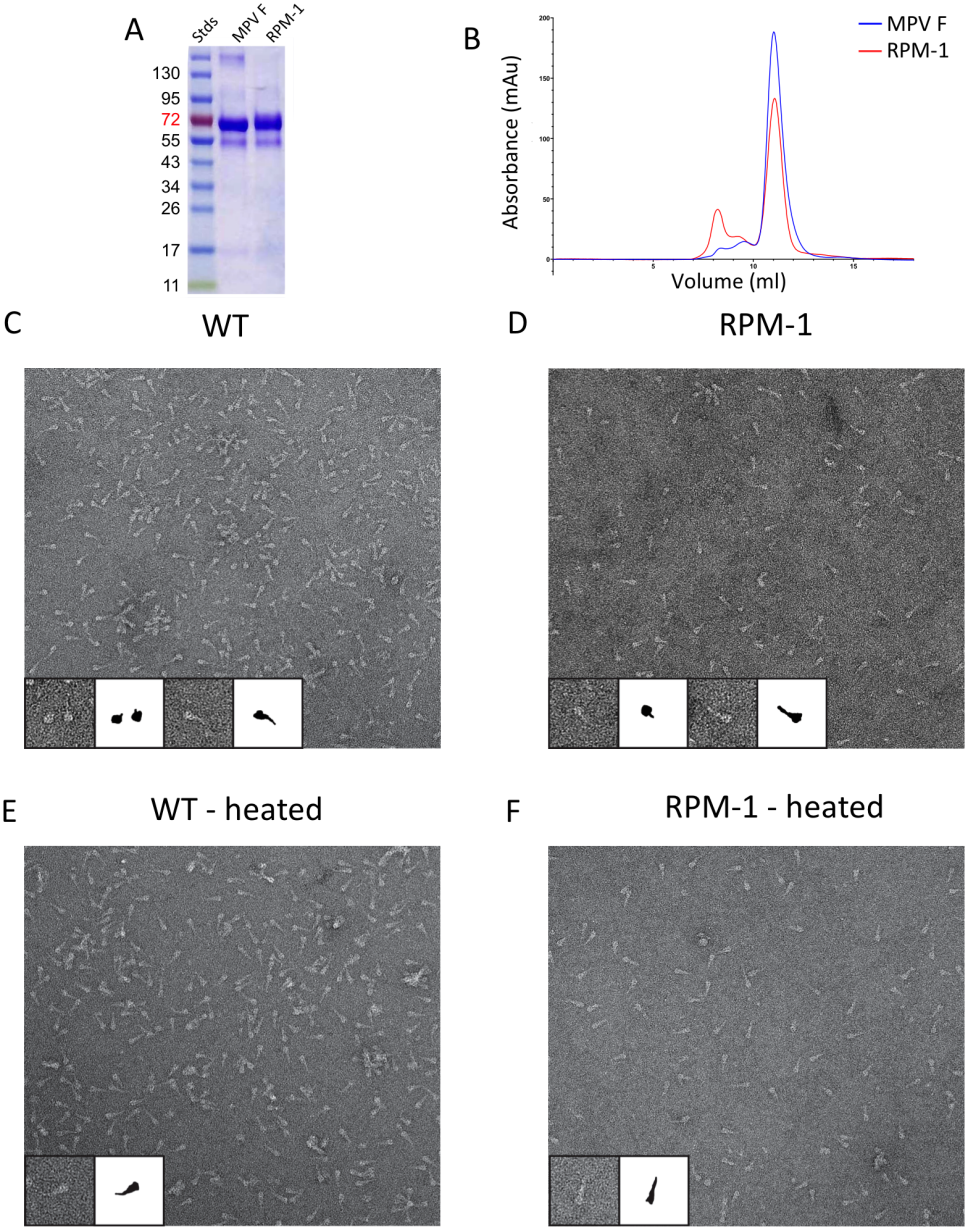
Purification and comparative analysis of wt and mutant MPV F protein. (a) SDS-PAGE analysis of purified wild-type HMPV F and the RPM-1 mutant. (b) Comparison of gel filtration traces of purified wild-type and RMP-1 MPV F proteins, demonstrating that they migrate similarly as trimers. (c, d) Negative-stain EM analysis of wild-type (c) and RPM-1 (d) proteins demonstrating that they form similar distributions of pre- and post-fusion conformations. (e, f) EM analysis of heat-treated wild-type (e) and RPM-1 (f) F proteins demonstrating that both proteins convert to a golf-tee like post-fusion conformation. In panels c-f, insets show higher magnification of selected F trimers along with schematic representations.

The wild-type and RPM-1 mutant were expressed as soluble, secreted proteins in 293F cells by transient transfection. The constructs include a C-terminal helical trimerization domain (GCNt) that we have shown previously to partially stabilize the pre-fusion soluble form of the MPV F protein as well as other paramyxovirus F proteins [14, 18, 32, 33]. The mutant protein expressed to similar levels as that of the wild-type protein and could be purified to homogeneity (Fig 2A). Both proteins migrated as trimers in gel filtration chromatography with elution volumes consistent with the soluble F trimer molecular weight (Fig 2B). The expression level of the RPM-1 mutant was similar to that of the wild-type protein, suggesting that there were no major defects in protein biosynthesis or folding caused by the epitope mutations.

We next examined the HMPV F proteins by negative-stain electron microscopy (Fig 2C and 2D). Both wild-type and mutant HMPV F proteins appeared primarily as discrete trimer assemblies. The EM analysis of the proteins is consistent with mixtures of both pre- and post-fusion forms being present to varying degrees in the preparations. We have shown previously that heating the F protein to 50°C can activate the refolding from the pre- to post-fusion conformation [32]. When the wild-type and RPM-1 F proteins were heated, EM images showed a predominance of golf-tee shaped trimers that are consistent with the refolding to the post-fusion state (Fig 2E and 2F). The data indicate that there are no significant issues in the folding, oligomerization or conformations of the F mutant, consistent with the surface location of the residues changed. Since motavizumab and palivizumab recognize both pre- and post-fusion RSV F, we anticipated that both antibodies would be able to bind to the recombinant RPM-1 mutant as well.

### The RPM-1 mutant but not wild-type HMPV F binds both DS-7 and palivizumab antibodies

To examine the ability of the RPM-1 mutants to bind palivizumab and DS-7 antibodies, we used a Biorad Proteon SPR instrument. DS-7 is an anti-HMPV F antibody whose epitope defines a novel neutralizing site on the F DI beta-sheet domain [14]. We anticipated that the RPM-1 mutant would retain its binding to DS-7 with wild-type properties, as no residues in that epitope were altered by the introduced mutations. Therefore, both DS-7 and palivizumab Fabs were used to test for binding with both wild-type and mutant MPV F proteins.

**Fig 3:**
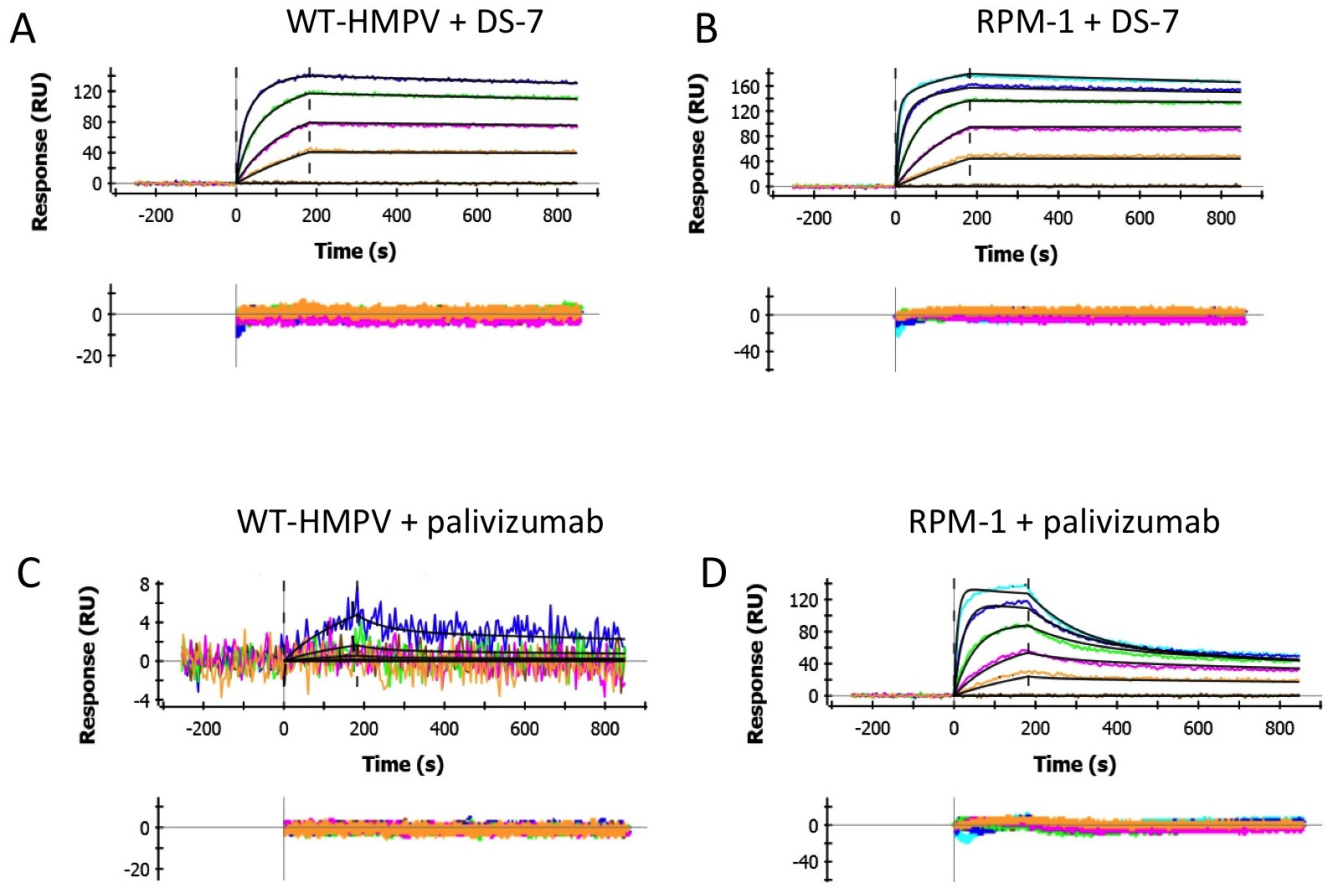
RPM-1, but not wild-type HMPV F, binds neutralizing antibodies to both RSV and HMPV. (a, b) Binding of the anti-HMPV F DS-7 antibody to wild-type (a) and RPM-1 (b) proteins. Both HMPV F proteins bound with similar affinity to the antibody demonstrating that the DS-7 epitope is not affected by the RPM-1 mutations. (c, d) Binding of palivizumab to wild-type (c) and RPM-1 (d) proteins. The wild-type HMPV F protein showed very weak potential binding palivizumab, while the RPM-1 mutant showed clear high affinity interactions with the antibody.

As anticipated, we observed that DS-7 was able to bind both proteins (Fig 3A and 3B). The best global fit to the kinetic traces was obtained using a bivalent analyte model. For both wild-type and mutant HMPV F, the kinetics and equilibrium constants were similar (Table 1). The overall equilibrium K_D_ was approximately 0.8 nM for the wild-type F and 2 nM for RPM-1. The kinetic rate constants for DS-7 binding also were similar (Table 1), with off-rates corresponding to a t_1/2_ of dissociation of 1 to 1.5 hours.

**Table 1:** Binding of antibodies to wt and RMP-1 MPV F.

Protein	Antibody	ka x 10^4^	kd x10^-4^	t_1/2_	K_D_
		(M^-1^s^-1^)	(s^-1^)	(min)	(nM)
**Wild-type HMPV F**					
	DS-7	14.7 ± .04	1.20 ± .02	96 ± 2	0.83 ± .02
	DS-7*	68.4 ± .24	1.97 ± .06	59 ± 2	0.30 ± .01
	palivizumab	-	-	-	>μM
**Heated wild-type HMPV F**					
	DS-7	9.57 ± .03	2.58 ± .06	45 ± 1	2.80 ± .06
	palivizumab	-	-	-	>μM
**RPM-1 F**					
	DS-7	9.1 ± .03	2.08 ± .07	55 ± 2	2.32 ± .07
	palivizumab	10.85 ± .06	41.8 ± .6	2.77 ± .04	38.8 ± 0.6
	motavizumab*	241.3 ± 0.4	0.18 ± .01	657 ± 20	7.11 ± .01 (pM)
**Heated RPM-1 F**					
	DS-7	22.4 ± .10	2.11 ± .07	55 ± 2	0.96 ± .03
	palivizumab	20.2 ± .24	67.7 ± 1.6	1.71 ± .04	34.0 ± .88

The mutant and wild-type proteins then were tested for palivizumab binding (Fig 3C and 3D). The wild-type HMPV protein showed very weak to no binding with the palivizumab Fab, with traces that were essentially within the noise of the experiment at the highest concentrations tested (Fig 3C). By contrast, palivizumab Fab binding to the RPM-1 mutant was robust and easily observed (Fig 3D), with maximal RUs for binding that were similar to those of DS-7, validating the successful grafting of the RSV epitope onto the HMPV F protein background with conformational fidelity. The overall equilibrium, K_D_ for palivizumab binding was 39 nM, with an off-rate of 4.2 x 10^−3^ s^-1^ corresponding to a t_1/2_ of ~3 minutes. By comparison, palivizumab binds to post-fusion RSV F with an affinity of ~1–4 nM [16, 17] and to a synthetic, scaffolded RSV epitope protein (MES-1) with an affinity of ~87 nM [30].

### Heat treatment of HMPV F does not affect neutralizing antibody binding

Heating of soluble, GCNt-stabilized F constructs converts the pre-fusion protein to its post-fusion form (Fig 2E and 2F). Since palivizumab has been shown to bind to both pre-and post-fusion RSV F, we sought to test whether heating the RPM-1 mutant would effect DS-7 or palivizumab Fab binding. The DS-7 epitope is largely conserved in pre-and post-fusion HMPV F, although there is the predicted potential for some binding interactions to occur within the pre-fusion HRB linker [14]. This possibility has not been directly tested by quantitatively examining the binding affinities to pre- or post-fusion HMPV F.

**Fig 4:**
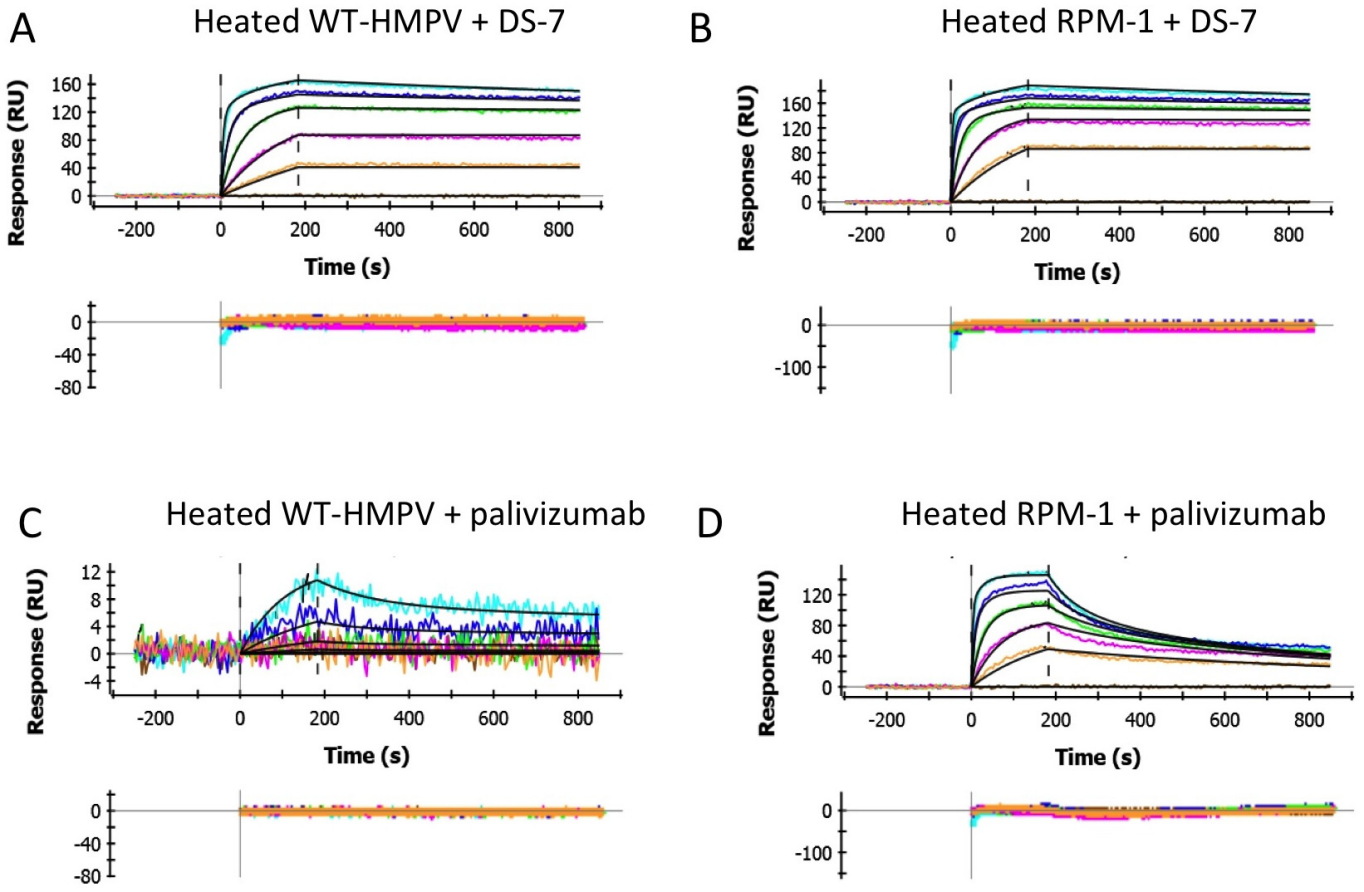
Heat treatment of wild-type and mutant RSV F does not affect binding of palivizumab and DS-7 neutralizing antibodies. (a, b) Binding of the anti-HMPV F DS-7 antibody to wild-type (a) and RPM-1 (b) proteins after heating to 50°C for 30 minutes. Both proteins bound with similar affinity to the antibody as the unheated F (Fig 3), demonstrating that the DS-7 epitope is not affected by conversion to the post-fusion conformation. (c, d) Binding of palivizumab to heat-treated wild-type (c) and RPM-1 (d) proteins. The wild-type HMPV F protein and RPM-1 mutant showed similar binding interactions with palivizumab as with the unheated F samples, indicating that concversion of pre- to post-fusion conformation had little effect on palivizumab antibody binding.

We heated HMPV F proteins to 50°C for 30 minutes and repeated the SPR binding experiments (Fig 4A and 4B). DS-7 bound to both wild-type and mutant RPM-1 similarly, and the equilibrium binding K_D_ values and rate constants were essentially identical to those of unheated F (Table 1), consistent with the expectation that the DS-7 epitope is not affected significantly by the pre- to post-fusion conformational change.

Binding of the palivizumab Fab also was tested, providing similar results to those with unheated F (Fig 4C and 4D). The heated HMPV F did exhibit slightly higher background binding to palivizumab (Fig 4C), although this binding remained a low-affinity interaction representing ~6% of the maximal RUs observed for the binding of the DS-7 Fab at the highest palivizumab concentrations tested. From these weak binding data, we estimated a cross-reactive binding affinity for the palivizumab-MPV F interaction to be >1,000-fold higher than that for RSV F. The heated RPM-1 mutant (Fig 4D) showed similar strong binding to palivizumab as unheated F, with K_D_ and rate constants collected in Table 1.

### Motavizumab binding affinity to the RPM-1 mutant parallels the high affinity difference observed for RSV F

Motavizumab is a re-engineered variant of palivizumab that exhibits ~100 fold higher binding affinity for RSV F [10]. Since the RPM-1 mutant design was based on the structure of the motavizumab:peptide complex [30], we anticipated that this affinity difference between palivizumab and motavizumab would be maintained by the chimeric mutant. Analysis of the motavizumab:peptide complex superimposed onto the wild-type RSV F structures further indicated that no additional contacts would be made outside of the helix-turn-helix region. However, within the HMPV F background, unanticipated additional contacts or steric conflicts outside of the epitope potentially could occur due to structural differences and thus impact motavizumab binding.

**Fig 5:**
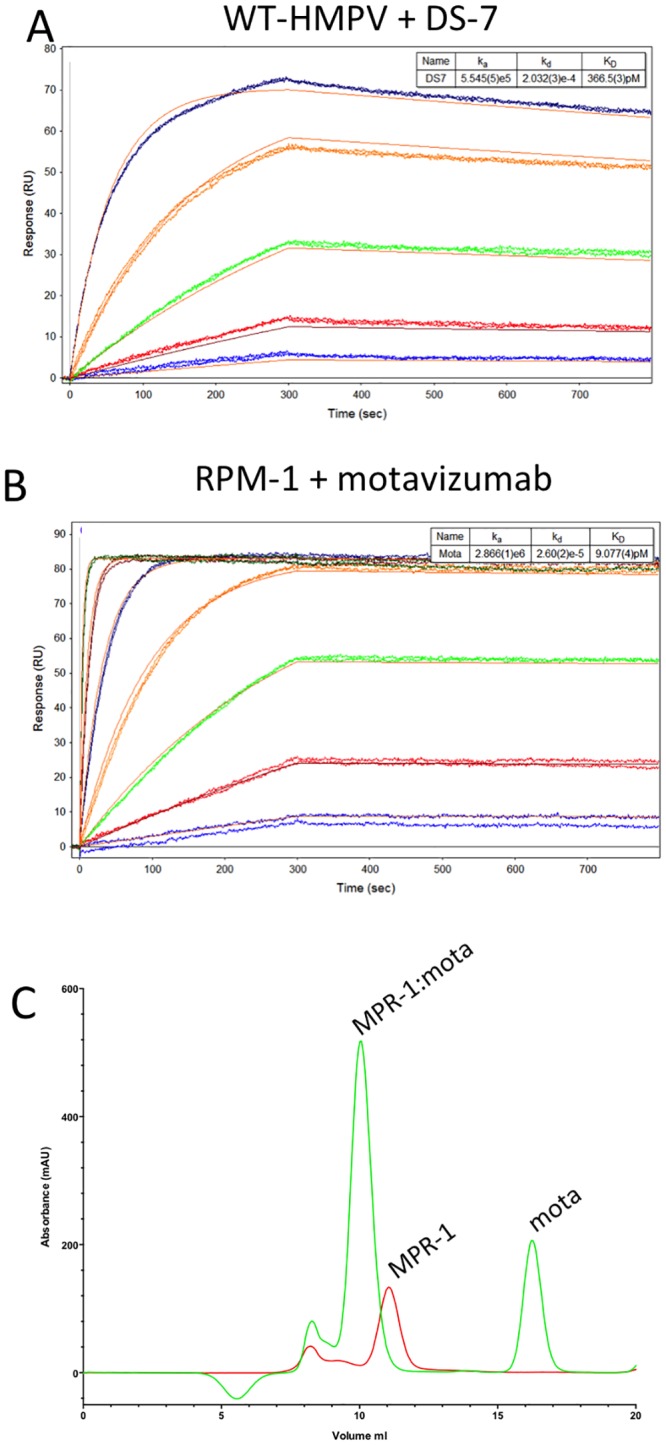
The HMPV F mutant bound motavizumab with higher affinity than palivizumab. Binding of the DS-7 Fab to wild-type (a) and motavizumab Fab RPM-1 (b) proteins using a BiOptix 404pi instrument. The wild-type HMPV F protein bound DS-7 with similar affinity as measured with the Biorad Proteon (Table 1). The RPM-1 mutant bound motavizumab with higher affinity than palivizumab. (c, d) Gel filtration analysis of the formation of RPM-1 complexes with palivizumab (c) and motavizumab (d) Fabs. The palivizumab complexes were unstable and dissociated during the gel filtration analysis, while motavizumab formed stable complexes consistent with three Fabs engaging a single RPM-1 trimer.

We tested the binding of DS-7 and motavizumab in parallel experiments using a BiOptix 404pi SPR instrument (Fig 5). DS-7 binding affinity to wt HMPV F was similar to that measured with the Biorad Proteon instrument, yielding a K_D_ of approximately 0.3 nM and comparable on/off rates (Table 1). Motavizumab Fab binding to the RPM-1 mutant showed dramatic affinity increases as compared to palivizumab (Fig 5). The measured K_D_ value was approximately 7 pM with an on-rate of ~2.4 x 10^6^ M^-1^s^-1^ and off-rate of ~1.8 x 10^−5^ s^-1^, corresponding to a t_1/2_ of >10 hours. This finding represents an approximately 1,000-fold increase in affinity relative to palivizumab and agrees well with affinity differences observed for the binding of palivizumab and motavizumab to wild-type RSV F and epitope scaffolded protein [17, 29, 30]. The high affinity binding of motavizumab further validates the grafting of the RSV F epitope onto the MPV F background, confirming that the underlying architecture of the MPV F hydrophobic core is preserved well enough to present the epitope surface residues in a closely native conformation to these two site II/site A antibodies.

Both palivizumab and motavizumab were used to make complexes with the RPM-1 mutant, which were analyzed by gel filtration chromatography. Palivizumab complexes did not yield a new peak of appropriate molecular weight, indicating that the complexes might dissociate during the course of the gel filtration experiment. By contrast, motavizumab formed stable complexes with RPM-1 with a new peak appearing earlier in the chromatogram eluting at a volume of 10 mls (Fig 5C). The shift in apparent molecular weight of the motavizumab:RPM-1 complexes is consistent with 3 Fabs binding to a single trimer, with a total predicted MW of ~330 kD. The observed instability of the palivizumab complexes and formation of stable motavizumab complexes is consistent with the different complex half lives measured by SPR (Table 1).

### Immunization of mice with RPM-1

To test the ability of the RPM-1 protein to induce antibody responses to the grafted RSV site A, we immunized BALB/c mice (6–8 weeks) with 100 μg of a mock control or each of 3 antigens in Titermax adjuvant. The antigens were MPV F pre-fusion/unheated protein, MPV F post-fusion/heated protein and the RPM-1 protein (post-fusion). Heat treatment (50°C, 30 min) of the MPV F-GCNt was used to convert all of the purified protein to the post-fusion form as we have described earlier. Using EM and functional antibody binding we have established that the RPM-1 protein is less stable than wt MPV F and rapidly converts to the post-fusion conformation. On day 28, the mice were boosted with 50 μg of antigen (or control). The terminal blood draw was conducted after an additional three weeks and the sera were tested in ELISA and neutralization assays.

**Fig 6:**
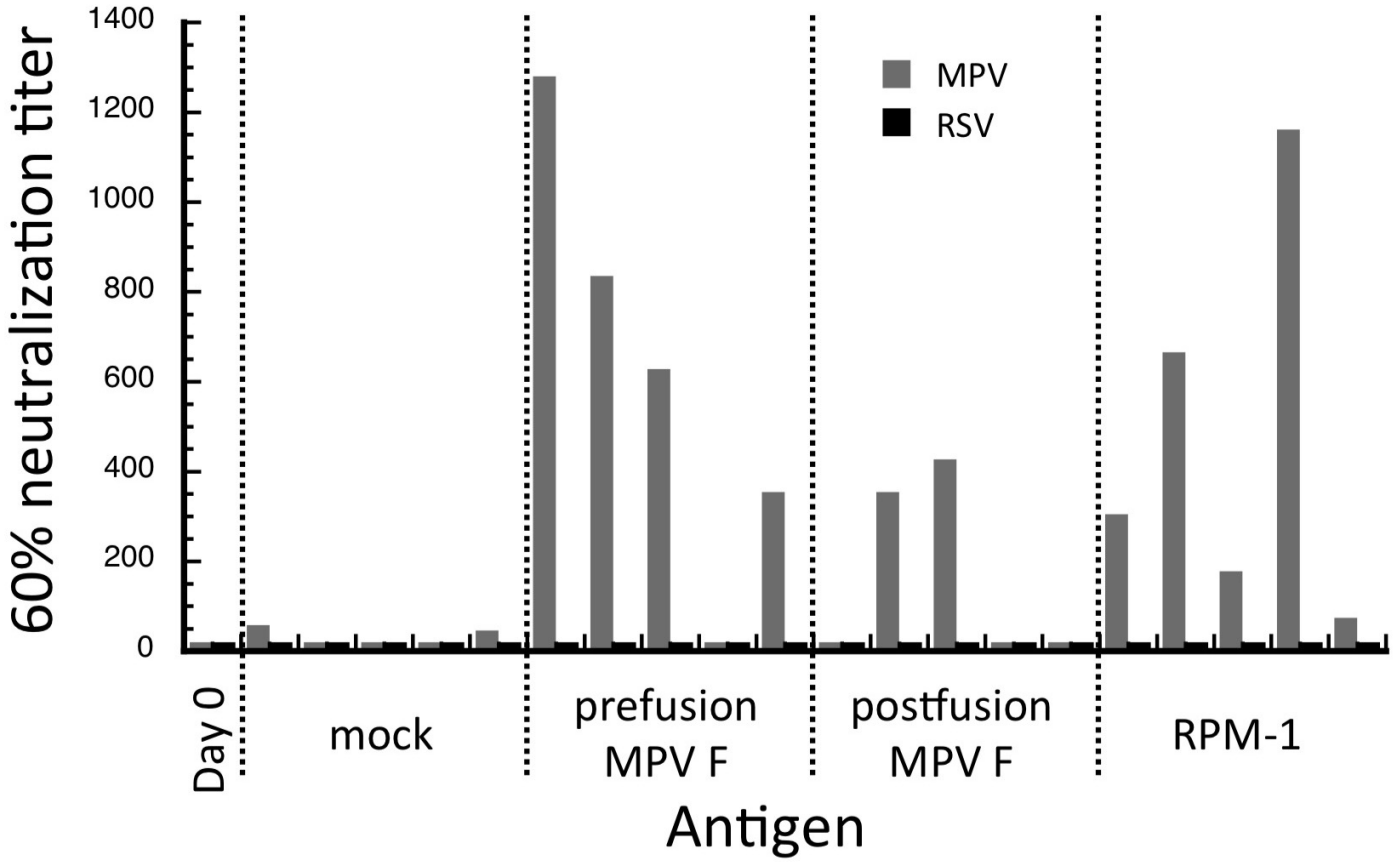
RSV and MPV neutralization assays. Data for each of 5 mice immunized with one of the three antigens (pre-fusion MPV F, post-fusion MPV F and RMP-1) is shown as the serum dilution required to obtain 60% neutralization. None of the mice showed neutralization of RSV, 4/5 immunized with pre-fusion F or RPM-1 showed MPV neutralization and 2/5 showed MPV neutralization with post-fusion MPV F.

All of the mice showed responses to the immunizing proteins, but no RSV-specific neutralizing activity was detected (Fig 6). By contrast, we observed robust MPV neutralizing activity in the sera from the majority of the MPV F and RPM-1 immunized mice, confirming that the RPM-1 mutant induces antibody responses to other MPV F epitopes (Fig 6). The sera also were tested in direct binding ELISAs (S1 Fig). Sera derived from the MPV F immunized mice showed no detectable binding to RSV F protein. However, sera from 1 out of the 5 mice immunized with RPM-1 did exhibit binding to RSV F (S1 Fig), suggesting that an RSV-specific response might be enhanced by changes to the immunization protocol, such as increasing the antigen dose, number of boosts or adjuvant. We note that in prior studies of a scaffolded site A epitope presented in the context of a helical structure, the resultant immune response in mice was also weak and increased with the number of antigen boosts [29]. In contrast, an immunoglobulin-based scaffolded site A epitope generated more robust RSV neutralizing responses [31]. The induction of robust neutralizing antibody responses to RSV glycoproteins provides strong protection against infection in animals [34] and is the goal of many current vaccine efforts [35]. The lack of a strong neutralizing antibody response in our RPM-1 immunized mice indicates that the presence of a single grafted epitope is unlikely to induce sufficient protection to virus challenge given the immunization protocol tested here. Overall, these data demonstrate that the RPM-1 chimera generates a robust nAb response to MPV in mice that is much stronger than the targeted site A-specific RSV response. This dual RSV/MPV specific antigen provides a starting point for further development of a single subunit vaccine for both viruses and for investigating the structural features needed to generate a potent RSV epitope-directed neutralizing response.

## Conclusions

Both RSV and HMPV represent major health problems worldwide, yet vaccine development efforts over many decades have not yet been successful [36, 37]. The need to balance safety and efficacy, especially in young children, as well as challenges in anticipating the induction of vaccine-induced disease represent significant barriers in RSV vaccine development. However, recently developed approaches appear promising and the potential for developing a pan-pneumovirus vaccine to both RSV and HMPV is attractive conceptually, as RSV and HMPV together are responsible for a majority of respiratory disease in children and the elderly after influenza virus. The prophylactic use of palivizumab in infants indicates that a sufficiently strong antibody response against a single epitope may be sufficient for the induction of protective immunity and epitope scaffolding approaches, initially pioneered for use in HIV vaccine design, have shown promise in this regard [29, 31].

The RSV site II/A epitope recognized by palivizumab and motavizumab represents an attractive target for epitope design and grafting, as it is largely defined by a linear peptide segment in RSV that forms a simple helix-turn-helix motif [19, 29, 30, 38]. Grafting of this motif onto a completely unrelated helical backbone yields novel proteins that are bound by palivizumab and motavizumab [29, 30] and here we demonstrated that similar grafting of the RSV epitope onto HMPV F also results in high affinity anti-RSV F antibody binding to the RPM-1 chimera. In preliminary experiments, we have shown that the RPM-1 chimera is useful for mapping the specificity and cross-reactivity of anti-RSV and anti-HMPV immune responses.

The observation that mice immunized with RPM-1 generated a neutralizing anti-MPV F antibody response but only a weak anti-RSV F response, is somewhat unexpected and provides insight into the comparative potency of the site A epitope relative to the remaining MPV F epitopes within the chimera. Previous studies with the RSV site A peptide grafted onto helical or Ig-domain scaffolds have demonstrated the possibility of generating site-specific, anti-RSV antibody responses [29, 31], although direct comparative studies between RPM-1 and these other antigens would be needed to assess their relative potencies in eliciting site A antibodies. For example, a helically-scaffolded site A antigen also did not induce strong anti-RSV F responses in mice, while neutralizing antibodies were more readily detected in macaques [29], consistent with our current observations with RPM-1. It may be that the site A peptide has intrinsic features that limit it’s ability to dominate an immune response relative to other F epitopes presented in the chimeric antigen. A potential intrinsic limitation may be the relative structural isolation of the raised helix-turn-helix motif relative to the surrounding F surface, which could limit the recruitment and maturation of high affinity antibodies relative to other epitopes. The limited site A response may additionally reflect the fact that this site represents only one of many potential epitopes on F and thus any site A specific response may be relatively diluted relative to MPV F specific surfaces. The grafting of additional RSV F epitopes onto MPV would likely balance the RSV and MPV responses and raises questions about which epitopes would be most valuable to graft into a chimeric protein. Structural information is currently available for multiple RSV F epitopes targeted by the D25 [15], AM14 [23] and 101F [39] antibodies. Grafting of the D25 epitope onto MPV F may be particularly interesting as this prefusion specific, DIII site contains glycosylation sites in MPV F [15] and grafting the RSV epitope would therefore have limited impact on MPV-specific sites by exposing this additional surface to immune recognition. Generating RSV/MPV F chimeras that display additional epitopes should result in a more balanced neutralizing antibody response to both viruses.

Many approaches could be taken in developing a pan-pneumovirus RSV/HMPV vaccine, including co-immunization with RSV and HMPV F antigens, designing separately scaffolded RSV/HMPV epitopes or developing immunization strategies to elicit rare, cross-protective antibodies. A chimeric antigen approach has been used successfully to elicit cross-protective responses to *N*. *meningitides* infection, using a synthetic antigen that presents the major variants of the factor H-binding protein (fHbp) [40, 41]. Chimeric RSV/MPV F antigens based on RPM-1 variants may therefore prove useful as novel vaccine candidates as well as for the further investigation of RSV and HMPV neutralizing antibody responses and their induction.

## Materials and Methods

### Expression and purification of MPV F and antibody proteins

HMPV F proteins were produced as previously described [14]. Briefly, the F gene from the HMPV B2 strain was cloned into the pcDNA3.1 expression vector with a full-length GCN4 trimerization (GCNT) domain in-frame with the heptad repeat of the C-terminal HRB^1,9^. The HMPV F expression plasmid was prepared using a Plasmid Mega Kit (Qiagen) and transfected into 293F cells (Invitrogen) at a density of 1.5 million cells/mL using polyethylenimine (PEI). Supernatants were harvested five days post-transfection by centrifugation (15 min at 8,000 x *g* at room temperature), filtered through 0.2 μm filters and dialyzed against 200 mM NaCl, 50 mM Na_2_HPO_4_ pH 7.4. The HMPV F protein was purified by Co^2+^ affinity chromatography (TALON Resin, BD Biosciences) and size exclusion chromatography using a Superdex-200 column equilibrated in 25 mM sodium phosphate, pH 7.4, and 100 mM NaCl. The purified protein was concentrated with Amicon Ultra centrifugal filters with a 10 kD molecular weight cut-off (Millipore), in a buffer containing 20 mM Tris (pH 8.0), 100 mM NaCl, 100 mM imidazole, 5 mM EDTA.

The RPM-1 mutant was prepared from the wild-type HMPV F construct by Mutagenex, with the following HMPV F mutations: A225S; A228L; V231I; S232N; Y233D; A238N; G239D; I241K; L245S; E246N. The RPM-1 mutant protein was expressed similarly to wild-type HMPV F in 293F cells, with supernatants harvested five days post-transfection. The HMPV F protein was purified by Co^2+^ affinity chromatography and size exclusion chromatography and concentrated as with wild-type HMPV F.

DS-7 Fab was expressed as previously described [14]. A double-gene construct encoding both heavy and light chain genes was transformed into DH5α *E*. *coli* cells (Invitrogen). Plasmid DNA was prepared using a Giga Kit (Qiagen) and transfected into 293F cells (Invitrogen) using PolyFect reagent (Qiagen) in a 10 liter WAVE bioreactor bag (GE). After one week, supernatant was purified over a gravity column with Capture Select resin (BAC B.V., The Netherlands) and eluted with citrate buffer. DS-7 Fab was concentrated with Amicon Ultra centrifugal filters with a 30 kD molecular weight cut-off (Millipore).

### Motavizumab Fab expression and purification

We synthesized cDNAs based on the heavy and light chain protein sequences of motavizumab that were optimized for mammalian cell expression (Genscript). The motavizumab heavy- and light-chain variable genes were cloned into the pEE6.4 and pEE12.4 vectors (Lonza), respectively, using the unique cloning sites. Purified plasmid DNA was co-transfected at a 1:1 heavy-light ratio into FreeStyle 293-F cells (Life Technologies) using 25 kDa linear polyethylenimine (PEI, Polysciences Inc.) transfection reagent at a ratio 2:1 of PEI to DNA. Antibody was purified and concentrated from supernatant on a protein G column (GE).

### Biosensor binding experiments

The palivizumab and DS-7 Fab binding experiments were performed on a ProteOn^™^ XPR36 instrument (Bio-Rad). Biotinylated antibodies were immobilized on a ProteOn NLC sensor chip surface through Biotin coupling at 80–300 response units (RU) and a blank surface without antibody was created for use as a reference. HMPV F protein was injected at a flow rate of 100 μl min^−1^, at concentrations of 500, 166.7, 55.5, 18.5, 6.2 or 0.0 nM. The running buffer was 20 mM NaPO_4_ pH 7.4, 100 mM NaCl, 10 mM Imidazole, 0.005% Tween 20. Data were processed using Proteon software and double referenced by subtraction of the blank surface and buffer-only injection before local fitting of the data (real-time parallel referencing). Binding curves were fit to a bivalent analyte model, to perform K_D_ calculations.

The motavizumab binding experiments were performed on a BiOptix 404pi^™^ SPR instrument, with DS-7 binding used as a parallel control. The wild-type and mutant HMPV F proteins were captured onto a Bioptix NiHC1000m sensor chip surface in channels 1 and 2 through their C-terminal His tag to a final level of 120 response units (RU). Blank control surfaces (channels 3 and 4) were generated by omitting F protein from the buffer for use as a parallel reference. Antibody Fab protein was injected at a flow rate of 100 μl min^−1^, at concentrations of 300, 100, 33.3, 11.1, 3.7, 1.23, 0.41, 0.14 or 0.0 nM. The running buffer consisted of 10 mM HEPES pH 7.2, 150 mM NaCl, 0.005% Tween 20, 50 μM EDTA. Before injecting the next sample concentration, channels 1 and 2 were regenerated by stripping the F protein and recapturing new F to the same target RU value. The data were processed using Bioptix software (Scrubber) and referenced by subtraction of the buffer-only injection. Binding curves were fit to a simple Langmuir model for K_D_ calculations.

### EM analysis of F proteins

Solutions of HMPV F were absorbed onto copper grids covered with carbon film that had been freshly glow discharged. Grids were stained with a 2% aqueous solution of uranyl formate, freshly prepared and filtered immediately prior to use. Grids were observed in a JEOL 1400 electron microscope operated at 120kV and images were acquired with a Gatan Ultrascan 4000. The pre-fusion HMPV F head region had a mean diameter of 7.48 nm (s.d. 1.1), with a range from 5.05–9.91 nm (n = 116).

### Immunization with RPM-1 and wt MPV F proteins

Female mice were used for the in vivo studies. The animals were cared for in an animal biosafety level 2 facility. The Vanderbilt University program is supported by a comprehensive Animal Care and Use Program (ACUP) that is registered with the United States Department of Agriculture (Registration #63-R-0005) and operates under Public Health Service Animal Welfare Assurance Statement #A3227-01. The Vanderbilt ACUP has been accredited by the Association for the Assessment and Accreditation of Laboratory Animal Care, International since 1967 (AAALAC file #000020) and most recently received “Continued Full Accreditation” on July 11, 2014. A comprehensive preventative medicine and veterinary care program was used that included daily observation of animals (including weekends) by animal care, veterinary, and research staff. Standard mouse chow and fresh water were provided *ad libitum*.

Five BALB/C mice were given an initial dose of 100 μg of (1) MPV F (pre-fusion) wild-type, (2) MPV F(post-fusion) wild-type, (3) RPM-1 Chimera or (4) Buffer control (Mock), with each protein delivered in Titermax. On day 28, the mice were boosted with 50 μg of antigen (or control). The terminal blood draw was conducted after an additional three weeks, and the sera were tested in ELISA and neutralization assays. The Vanderbilt Institutional Animal Care and Use Committee (IACUC) reviewed and approved this research under Protocol ID#: M/13/046. No adverse outcomes were observed.

## Supporting Information

S1 Fig anti-RSV F and anti-HMPV F ELISA titers. Antigens (pre-/post-fusion RSV F, pre-/post-fusion MPV F and RPM-1) were coated onto plates. The serum from 5 mice immunized with pre-fusion MPV F (MPV pre), post-fusion MPV F (MPV post) and RPM-1 was diluted 1:100–1:32,000 and binding to the antigens was detected using a secondary anti-mouse antibody (Methods).
(PDF)
