# Supplementary material for: A Chimeric Pneumovirus Fusion Protein Carrying Neutralizing Epitopes of Both MPV and RSV
Source: PLoS One. 2016 May 25;11(5):e0155917. doi: 10.1371/journal.pone.0155917 (PMC4880302; doi:10.1371/journal.pone.0155917)
Supplement: S1 Fig — Antigens (pre-/post-fusion RSV F, pre-/post-fusion MPV F and RPM-1) were coated onto plates. The serum from 5 mice immunized with pre-fusion MPV F (MPV pre), post-fusion MPV F (MPV post) and RPM-1 was diluted 1:100–1:32,000 and binding to the antigens was detected using a secondary anti-mouse antibody (Methods). (PDF) [file pone.0155917.s001.pdf]

Supplementary Figure 1. RSV and MPV F ELISA titers. Antigens (pre-/post-fusion RSV F, pre-/post-fusion MPV F and RPM-1) were coated onto plates. The serum from 5 mice immunized with pre-fusion MPV F (MPV pre), post-fusion MPV F (MPV post) and RPM-1 was diluted 1:100-1:32,000 and binding to the antigens was detected using a secondary anti-mouse antibody (Methods).

RSV F post coated

Mouse --> / Dilution factor	MPV pre (1)	MPV pre (2)	MPV pre (3)	MPV pre (4)	MPV pre (5)
100	0.04	0.00	0.07	0.00	-0.01
500	0.01	-0.01	0.01	-0.01	-0.01
1000	0.01	-0.02	0.01	0.00	-0.02
2000	0.00	-0.01	0.02	0.00	-0.01
4000	-0.01	0.16	0.23	-0.01	-0.02
8000	0.00	0.01	0.00	-0.01	-0.01
16000	0.01	0.01	0.00	0.00	0.01
32000	0.03	0.02	0.02	0.02	0.01

RSV post F coated

Mouse --> / Dilution factor	MPV post (1)	MPV post (2)	MPV post (3)	MPV post (4)	MPV post (5)
100	0.01	0.00	-0.02	-0.01	0.03
500	0.01	0.02	0.00	0.01	0.05
1000	0.00	0.00	0.04	0.01	-0.01
2000	0.00	0.00	-0.01	0.00	0.00
4000	0.02	-0.01	0.00	-0.01	0.00
8000	0.01	0.00	-0.01	0.00	0.00
16000	0.03	0.00	0.01	0.01	0.02
32000	0.02	0.00	0.00	0.01	0.00

RSV F post coated

Mouse --> / Dilution factor	RPM-1 (1)	RPM-1 (2)	RPM-1 (3)	RPM-1 (4)	RPM-1 (5)
100	-0.01	0.39	0.01	0.04	0.05
500	0.00	0.12	0.00	0.01	0.00
1000	0.00	0.04	-0.01	-0.01	-0.02
2000	0.01	0.01	0.00	-0.01	-0.01
4000	0.00	0.00	-0.01	-0.01	-0.02
8000	0.00	0.01	-0.01	0.00	0.00
16000	0.00	0.00	0.00	0.00	0.00
32000	0.01	0.00	0.01	0.12	0.00

RSV F pre coated

Mouse --> / Dilution factor	MPV pre (1)	MPV pre (2)	MPV pre (3)	MPV pre (4)	MPV pre (5)
100	0.01	0.00	0.00	0.00	0.01
500	0.00	0.00	0.00	0.01	0.00
1000	0.00	0.00	0.00	0.00	0.00
2000	0.00	0.00	0.00	0.00	0.00
4000	-0.01	-0.01	-0.01	0.00	-0.01
8000	0.00	-0.01	0.00	0.00	0.00
16000	-0.01	-0.01	-0.01	0.00	-0.01
32000	0.00	0.00	0.00	0.00	0.00

RSV pre F coated

Mouse --> / Dilution factor	MPV post (1)	MPV post (2)	MPV post (3)	MPV post (4)	MPV post (5)
100	0.01	0.00	0.00	0.00	0.01
500	0.00	0.00	0.00	0.01	0.00
1000	0.00	0.00	0.00	0.00	0.00
2000	0.01	0.01	0.01	0.01	0.01
4000	0.00	0.00	0.00	0.00	0.00
8000	0.00	0.00	0.00	0.00	0.00
16000	0.01	0.00	0.00	0.00	0.00
32000	0.02	0.01	0.01	0.01	0.01

RSV pre F coated

Mouse --> / Dilution factor	RPM-1 (1)	RPM-1 (2)	RPM-1 (3)	RPM-1 (4)	RPM-1 (5)
100	0.00	0.12	0.00	0.01	0.05
500	0.01	0.04	0.00	0.01	0.01
1000	0.00	0.02	0.00	0.01	0.00
2000	0.01	0.02	0.01	0.02	0.01
4000	0.00	0.01	0.00	0.00	-0.01
8000	0.00	0.01	0.01	0.00	0.00
16000	0.00	0.01	0.00	0.00	0.00
32000	0.01	0.02	0.01	0.02	0.01

MPV pre coated

Mouse --> / Dilution factor	MPV pre (1)	MPV pre (2)	MPV pre (3)	MPV pre (4)	MPV pre (5)
100	1.2	0.75275	0.82325	0.6065	0.98225
500	0.916	0.306	0.299	0.17825	0.49825
1000	0.6795	0.13175	0.141	0.09	0.3175
2000	0.418	0.07125	0.07	0.045	0.172
4000	0.218	0.03225	0.025	0.02075	0.069
8000	0.0925	0.016	0.0175	0.011	0.0295
16000	0.04175	0.01275	0.01175	0.00525	0.01725
32000	0.0315	0.0195	0.02125	0.01825	0.015

MPV pre F coated

Mouse --> / Dilution factor	MPV post (1)	MPV post (2)	MPV post (3)	MPV post (4)	MPV post (5)
100	0.49	0.49	0.68	0.38	0.88
500	0.19	0.17	0.26	0.15	0.30
1000	0.19	0.12	0.17	0.08	0.14
2000	0.22	0.13	0.16	0.09	0.11
4000	0.20	0.16	0.09	0.08	0.11
8000	0.16	0.11	0.05	0.06	0.05
16000	0.13	0.09	0.09	0.08	0.09
32000	0.21	0.33	0.15	0.16	0.16

MPV pre F coated

Mouse --> / Dilution factor	RPM-1 (1)	RPM-1 (2)	RPM-1 (3)	RPM-1 (4)	RPM-1 (5)
100	0.74	1.18	0.79	1.07	1.03
500	0.29	0.95	0.33	0.56	0.68
1000	0.20	0.75	0.20	0.36	0.46
2000	0.10	0.45	0.18	0.27	0.27
4000	0.08	0.25	0.09	0.15	0.16
8000	0.05	0.14	0.07	0.07	0.08
16000	0.07	0.09	0.05	0.08	0.08
32000	0.16	0.17	0.08	0.11	0.10

MPV post F coated

Mouse --> / Dilution factor	MPV pre (1)	MPV pre (2)	MPV pre (3)	MPV pre (4)	MPV pre (5)
100	1.2065	0.70025	0.78875	0.58825	0.934
500	0.89775	0.2605	0.28475	0.1715	0.556
1000	0.642	0.13125	0.128	0.08075	0.3065
2000	0.36225	0.0575	0.0615	0.041	0.142
4000	0.18275	0.02075	0.02225	0.01675	0.058
8000	0.07875	0.008	0.0095	0.009	0.0285
16000	0.027	-0.00275	-0.00125	0.0035	0.00825
32000	0.01575	0.00175	0.0025	0.006	0.0045

MPV post F coated

Mouse --> / Dilution factor	MPV post (1)	MPV post (2)	MPV post (3)	MPV post (4)	MPV post (5)
100	0.30	0.31	0.47	0.26	0.63
500	0.09	0.09	0.18	0.07	0.21
1000	0.04	0.03	0.07	0.03	0.09
2000	0.06	0.04	0.05	0.03	0.06
4000	0.01	0.01	0.02	0.01	0.03
8000	0.01	0.01	0.01	0.01	0.02
16000	0.01	0.01	0.01	0.01	0.01
32000	0.03	0.02	0.02	0.02	0.04

MPV post F coated

Mouse --> / Dilution factor	RPM-1 (1)	RPM-1 (2)	RPM-1 (3)	RPM-1 (4)	RPM-1 (5)
100	0.58	1.01	0.71	0.92	1.02
500	0.23	0.80	0.26	0.43	0.68
1000	0.12	0.56	0.13	0.30	0.37
2000	0.09	0.41	0.09	0.16	0.25
4000	0.05	0.20	0.04	0.08	0.11
8000	0.03	0.10	0.03	0.04	0.06
16000	0.01	0.05	0.02	0.03	0.02
32000	0.03	0.06	0.04	0.03	0.03

RPM-1 coated

Mouse --> / Dilution factor	MPV pre (1)	MPV pre (2)	MPV pre (3)	MPV pre (4)	MPV pre (5)
100	1.33775	0.66925	0.69925	0.54275	1.0445
500	0.9425	0.26625	0.2175	0.147	0.532
1000	0.676	0.1175	0.11075	0.07975	0.2875
2000	0.40375	0.06075	0.056	0.05025	0.16825
4000	0.19675	0.0325	0.02725	0.0225	0.0685
8000	0.08275	0.016	0.0165	0.01175	0.0335
16000	0.0385	0.01275	0.01225	0.00425	0.0175
32000	0.033	0.02175	0.02	0.02025	0.0195

RPM-1 coated

Mouse --> / Dilution factor	MPV post (1)	MPV post (2)	MPV post (3)	MPV post (4)	MPV post (5)
100	0.29	0.38	0.61	0.24	0.78
500	0.06	0.07	0.17	0.06	0.21
1000	0.02	0.03	0.07	0.02	0.09
2000	0.01	0.01	0.03	0.01	0.04
4000	0.00	0.00	0.01	0.00	0.02
8000	0.00	0.00	0.00	0.00	0.01
16000	0.00	0.00	0.00	0.00	0.00
32000	0.01	0.00	0.00	0.00	0.01

RPM-1 coated

Mouse --> / Dilution factor	RPM-1 (1)	RPM-1 (2)	RPM-1 (3)	RPM-1 (4)	RPM-1 (5)
100	0.80	1.24	0.87	1.06	1.22
500	0.32	1.10	0.33	0.59	0.73
1000	0.15	0.76	0.16	0.32	0.45
2000	0.06	0.48	0.08	0.17	0.27
4000	0.03	0.26	0.03	0.07	0.11
8000	0.01	0.12	0.01	0.04	0.05
16000	0.00	0.05	0.00	0.02	0.02
32000	0.01	0.03	0.01	0.01	0.01

Supplemental Figure 1
